# Stanniocalcin-2 contributes to mesenchymal stromal cells attenuating murine contact hypersensitivity mainly via reducing CD8^+^ Tc1 cells

**DOI:** 10.1038/s41419-018-0614-x

**Published:** 2018-05-10

**Authors:** Xiaoyong Chen, Qiuli Liu, Weijun Huang, Chuang Cai, Wenjie Xia, Yanwen Peng, Shuwei Zheng, Gang Li, Yan Xu, Jiancheng Wang, Chang Liu, Xiaoran Zhang, Li Huang, Andy Peng Xiang, Qi Zhang

**Affiliations:** 1 The Biotherapy Center, the Third Affiliated Hospital, Zhongshan School of Medicine, Sun Yat-Sen University, 510630 Guangzhou, China; 2 Center for Stem Cell Biology and Tissue Engineering, Key Laboratory for Stem Cells and Tissue Engineering, Ministry of Education, Sun Yat-Sen University, 510080 Guangzhou, China; 3 Department of Pathophysiology, Zhongshan School of Medicine, Sun Yat-Sen University, 510080 Guangzhou, China; 4 Institute of Blood Transfusion, Guangzhou Blood Centre, 510095 Guangzhou, China; 5 Department of Biochemistry, Zhongshan Medical School, Sun Yat-Sen University, 510080 Guangzhou, China; 6 Guangdong Provincial Key Laboratory of Liver Disease Research, Guangzhou, China

## Abstract

Mesenchymal stromal cells (MSCs) have been demonstrated to ameliorate allergic contact dermatitis (ACD), a typical T-cell-mediated disorder. However, the underlying mechanisms behind the MSC-based treatment for ACD have not yet been fully elucidated. The stanniocalcins (STCs) comprise a family of secreted glycoprotein hormones that act as important anti-inflammatory proteins. Here, we investigated the roles of STCs in MSC-mediated T-cell suppression and their potential role in the MSC-based treatment for ACD. Gene expression profiling revealed that STC2, but not STC1, was highly expressed in MSCs. STC2 knockdown in MSCs significantly impaired their effects in reducing TNF-α- and IFN-γ-producing CD8^+^ T cells. Importantly, silencing the STC2 expression in MSCs abated their therapeutic effect on contact hypersensitivity (CHS) in mice, mainly restoring the generation and infiltration of IFN-γ-producing CD8^+^ T cells (Tc1 cells). Mechanistically, STC2 co-localized with heme oxygenase 1 (HO-1) in MSCs, and contributed to MSC-mediated reduction of CD8^+^ Tc1 cells via regulating HO-1 activity. Together, these findings newly identify STC2 as the first stanniocalcin responsible for mediating the immunomodulatory effects of MSCs on allogeneic T cells and STC2 contribute to MSC-based treatment for ACD mainly via reducing the CD8^+^ Tc1 cells.

## Introduction

Allergic contact dermatitis (ACD) is an inflammatory skin condition manifest as an allergic response caused by contact with immune-stimulating substances. Although there have been significant advances in the medical treatment of this disease, patients that are unresponsive to topical steroids or systemic immunosuppressant still have few therapeutic options^1,2^. ACD is a typical T-cell-mediated disorder, and the CD8^+^ effector T lymphocytes are likely the predominant effector population in ACD, especially the CD8^+^ T cytotoxic type I (Tc1) cells^3,4^. Mounting evidences have also showed that CD8^+^ T cells have a crucial effector role in murine contact hypersensitivity (CHS)^5–7^, the animal model of ACD. Thus, by targeting CD8^+^ T cells, we can hamper allergic responses in skin hypersensitivity^8^.

Mesenchymal stromal cells (MSCs), a multipotent stromal cell subset that can differentiate into osteoblasts, adipocytes, and chondrocytes^9^, have shown promise in preclinical and clinical therapies for a variety of T-cell-mediated diseases, largely due to their immunomodulatory effects on T cells. MSCs could suppress T-cell activation, inhibit T-cell proliferation, and reduce their secretions of pro-inflammatory cytokines^10,11^. MSCs reportedly inhibit both CD4^+^ T helper (Th) cells and CD8^+^ cytotoxic T lymphocytes via direct and/or indirect actions^12^. Recent preclinical and clinical studies demonstrated that MSCs are becoming a promising therapeutic option for ACD^13,14^. However, the underlying mechanisms behind the MSC-based treatment for ACD have not yet been fully elucidated.

Various soluble molecules have been implicated in the MSC-mediated inhibition of T cells, including transforming growth factor-β (TGF-β), hepatocyte growth factor, indoleamine-2,3-dioxygenase, prostaglandin E2, heme oxygenase-1 (HO-1) and HLA-G5^10,11,15^. However, the single blockade of any of the above-listed molecules failed to completely abrogate the immunosuppressive functions of MSCs, indicating that other important mediators remain to be identified.

The stanniocalcin (STC) family consists of two proteins, STC1 and STC2, which are expressed in various human tissues^16^, such as pancreas, spleen, kidney, and skeletal muscle. Numerous studies have examined STC1 and STC2 in the tumor microenvironment, where they have positive effects on tumor migration and invasion^17,18^. Clinically, STC2 has been proposed to be a biomarker for various cancers, in association with the formation of tumor neovascularization^19,20^. Importantly, the STCs have been shown to be important naturally occurring anti-inflammatory proteins^21,22^. STC1 exerts its anti-inflammatory effects by inducing uncoupling proteins and thus reducing oxidative stress^23^, and it reportedly counteracts LPS-induced lung injury by inhibiting the inflammatory cascade and inducing antioxidant and antiapoptotic mechanisms^24^. STC2, which is a homolog of STC1, is a stress-responsive protein that may be targeted by the oxidative stress response to protect cells from apoptosis. Functionally, STC2 has been associated with the unfolded protein response^25^, and has been shown to downregulate the TNF-α and IL-1β in LPS-stimulated BV2 cells^26^. Recently, MSC-derived STC1 was demonstrated to promote the survival of lung cancer cells^27^, and STC2 has been shown to critically enhance MSC survival. However, the potential involvement of the STCs in the immunomodulatory activities of MSCs has not yet been explored in detail. Here, we investigated the potential involvement of the STCs in MSC-mediated T-cell suppression and their potential role in the MSC-based treatment for T-cell-mediated ACD.

## Results

### STC2 is highly expressed in human MSCs

**Fig. 1:**
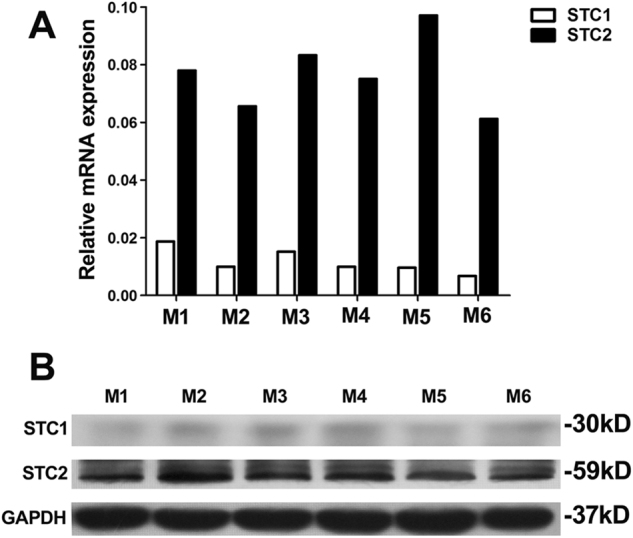
STC2 is highly expressed in human MSCs. The expressions of STCs were evaluated in hMSCs from six donors. **a** The relative mRNA expression levels of STC1 and STC2 were analyzed by qPCR and normalized with reference GAPDH controls. **b** Western blot analysis of STC1 and STC2 expression in hMSCs

Human MSCs were isolated and characterized as described in the Materials and Methods section. We used RT-PCR to detect the expression of mRNAs for STC1 and STC2 in MSCs. Our results showed that STC2 was highly expressed in human MSCs, whereas STC1 showed relatively low-level expression (about one eighth that of STC2) (Fig. 1a). A similar trend was observed at the protein level, far less STC1 than STC2 existed in six donors’ MSCs at the same passage (Fig. 1b). Due to the relatively abundant expression of STC2 in MSCs, we investigated the potential involvement of STC2 in mediating the immunomodulatory effect of MSCs on allogeneic T cells.

### Downregulation of STC2 does not change the characteristics of MSCs

To analyze whether MSC-expressed STC2 contributes to the immunomodulatory properties of these cells, we used RNA interference to deplete STC2 in cultured MSCs. RT-PCR showed that the mRNA expression of STC2 was 80% lower in knockdown cells (MSC^shSTC2^) compared with control-transfected MSCs (MSC^shNTC^) (Fig. S1A), and western blotting demonstrated that the STC2 protein was almost undetectable in MSC^shSTC2^ lysates (Fig. S1B).

We then examined the effect of STC2 knockdown on the characteristics of our MSCs. The surface markers, CD29, CD44, CD73, CD90, CD105, and CD166, were detected at similar levels on MSC^shNTC^ and MSC^shSTC2^, whereas both cell types lacked CD34 and CD45 (Fig. S2A). MSC^shNTC^ and MSC^shSTC2^ were fibroblast-like spindle cells, and showed the ability to undergo osteogenic and adipogenic differentiation were retained in MSC^shNTC^ and MSC^shSTC2^ (Fig. S2B). These results indicate that neither lentiviral infection nor STC2 knockdown influenced the phenotypic makers, shape, or differentiation potential of our MSCs.

### MSC-derived STC2 tended to reduce the CD8^+^ effector T cells, mainly the IFN-γ-producing Tc1 cells

**Fig. 2:**
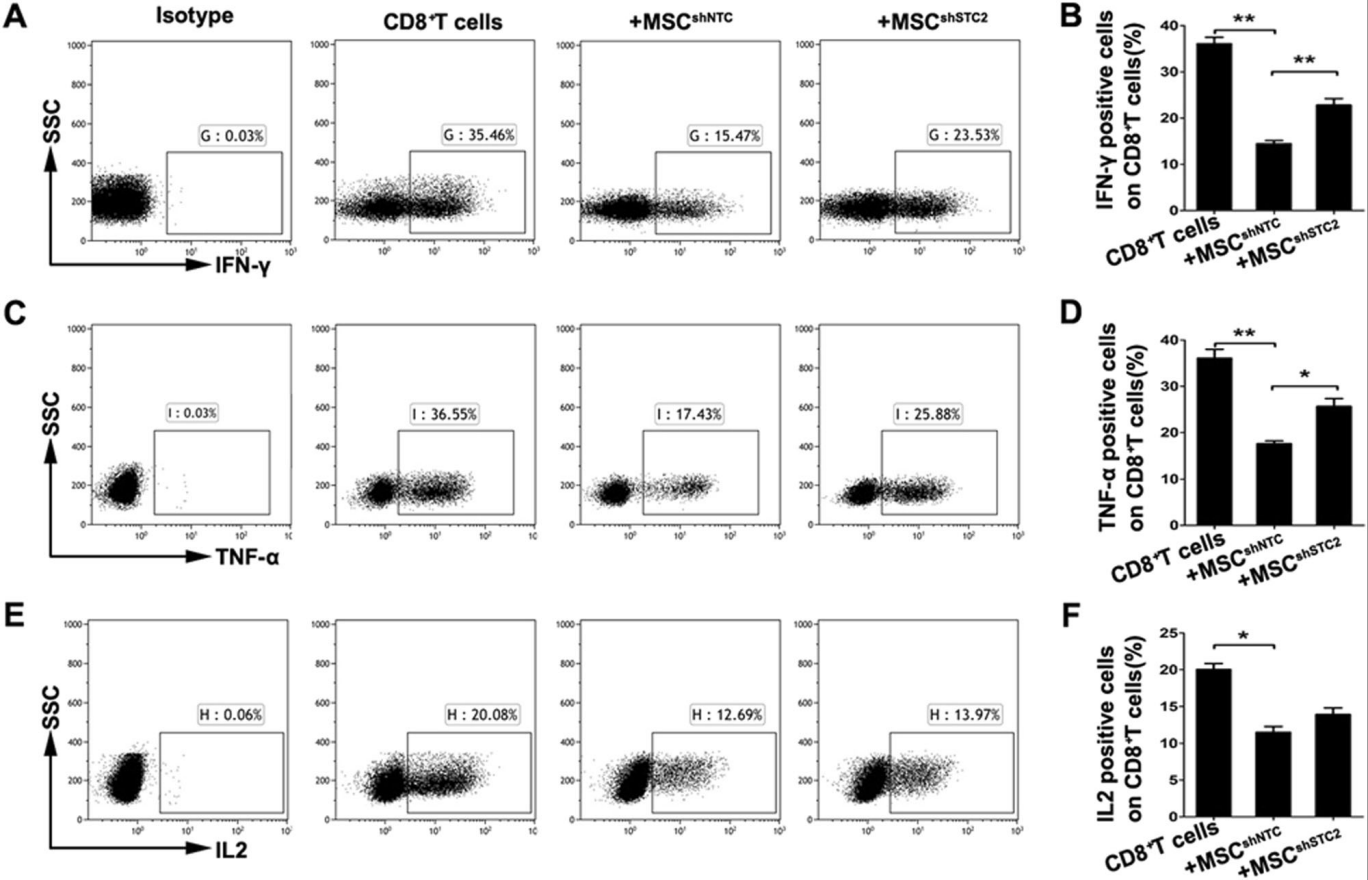
MSCs downregulate the pro-inflammatory cytokine-producing CD8^+^ T cells partially through STC2 in vitro. CD3^+^CD8^+^ T cells were co-cultured with MSCs for 3 days, and flow cytometry was used to assess IFN-γ, TNF-α, and IL-2 production. **a** Representative plots of alteration in percentages of IFN-γ-producing CD8^+^ T cells, and bar chart showed the quantification (**b**). **c** Representative plots of alteration in percentages of TNF-α-producing CD8^+^ T cells, and bar chart showed the quantification (**d**). **e** Representative plots of alteration in percentages of IL-2-producing CD8^+^ T cells, and bar chart showed the quantification (**f**). Data are presented as mean ± SEM (*n* = 4). Statistically significant differences are indicated as follows: *p < 0.05 and **p < 0.01

To investigate whether STC2 contributed to the immunomodulatory properties of MSCs, we examined the effects of MSC^shNTC^ or MSC^shSTC2^ on the production of pro-inflammatory cytokines from T cells. Our results revealed that co-culture with MSC^shNTC^ significantly reduced the productions of IFN-γ, TNF-α, and IL-2 by CD3^+^, CD4^+^, and CD8^+^ T cells, and that knockdown of STC2 decreased the ability of MSCs to suppress the T-cell-mediated productions of IFN-γ and TNF-α, but not IL-2 (Fig. S3, Fig. S4, Fig. 2). These effects were moderate in CD3^+^ (Figs S3) and CD4^+^ T cells (Fig. S4), but were dramatic in CD8^+^ T cells. As shown in Fig. 2, the productions of IFN-γ and TNF-α were significantly inhibited in MSC^shNTC^ co-cultured CD8^+^ T cells compared to mono-cultured CD8^+^ T cells. Co-culture with MSC^shSTC2^ yielded significantly more IFN-γ- or TNF-α-producing CD8^+^ T cells compared to MSC^shNTC^ co-cultures. Taken together, these results suggest that MSC-derived STC2 probably prefer to reduce pro-inflammatory cytokine producing CD8^+^ effector T cells, especially the IFN-γ-producing CD8^+^ T cells, known as CD8^+^ Tc1 cells.

### MSC-derived STC2 is negligible in MSC-mediated T-cell proliferation inhibition or Treg cell induction

As MSCs are known to inhibit the proliferation of T cells^11,28^, we analyzed whether STC2 could contribute to this effect. CFSE-labeled T cells were co-cultured with MSCs in the presence of PHA for 72 h, and T-cell proliferation was evaluated by CFSE dilution and flow cytometry. As shown in Fig. S5, MSC^shNTC^ robustly inhibited T-cell proliferation by approximately 70% compared to mono-cultured T cells. MSC^shSTC2^ showed a similar level of anti-proliferative activity, indicating that STC2 does not contribute to the MSC-mediated inhibition of T-cell proliferation.

Since MSCs have been reported to recruit and induce regulatory T cells (Tregs)^10,11^, we then investigated whether MSC-derived STC2 could contribute to this process. CD4^+^CD25^−^ T cells were isolated, and the percentages of Tregs were compared between T-cell mono-cultures and MSC/T cell co-cultures. The proportion of CD4^+^CD25^+^Foxp3^+^ Tregs was significantly increased in the MSC^shNTC^/T cultures compared to mono-cultured T cells; however, there was no difference between the MSC^shNTC^/T and MSC^shSTC2^/T groups (Fig. S6), suggesting that MSC-derived STC2 may not play a role in the induction of Tregs among T cells.

### MSCs ameliorate contact hypersensitivity in mice through STC2 in vivo

**Fig. 3:**
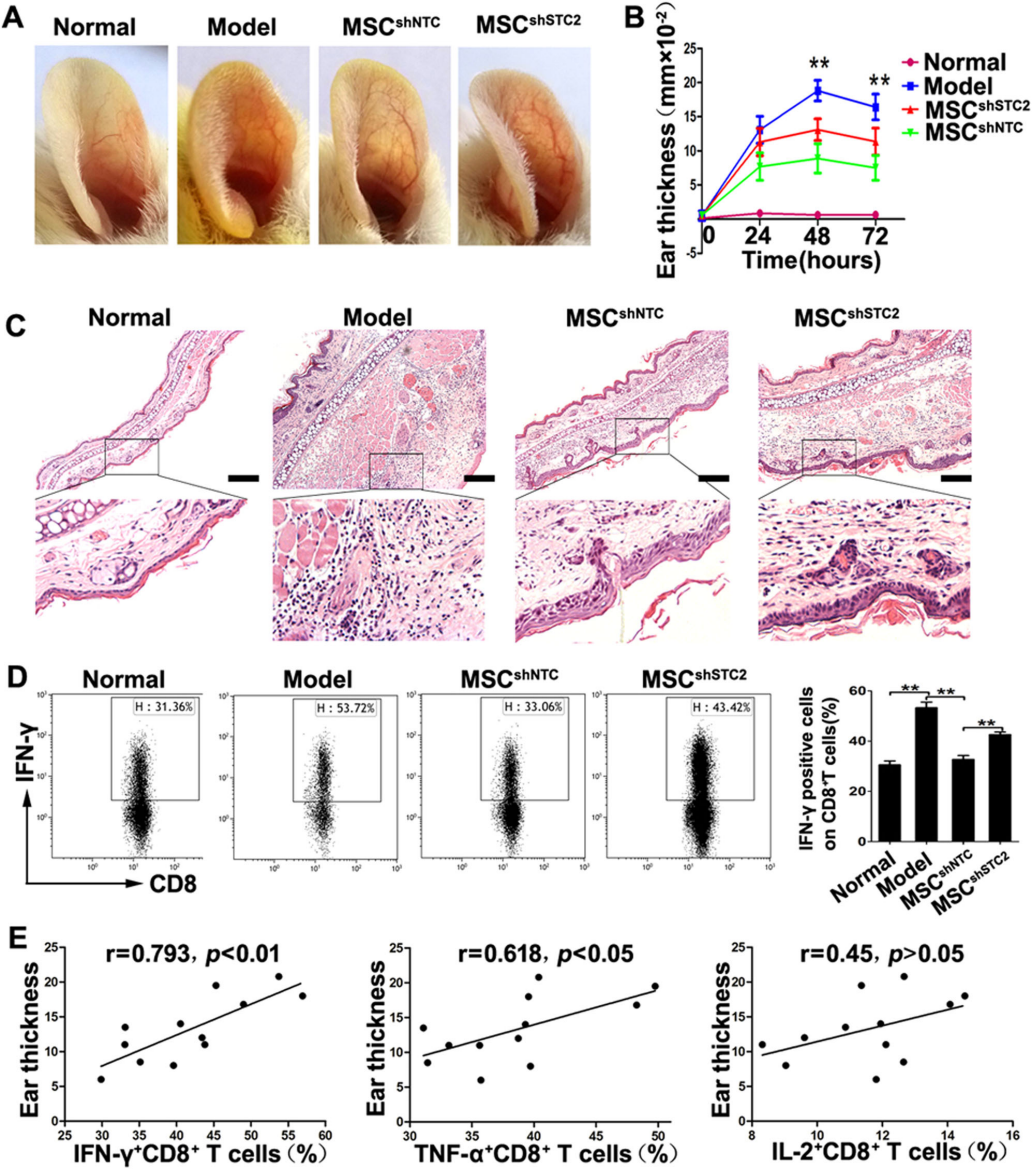
MSCs attenuate CHS partially through the STC2-dependent CD8^+^ Tc1 cells reduction in cervical lymph nodes. In mice, CHS reactions were induced with 0.5% DNFB in acetone/olive oil (4:1). MSC^shNTC^ or MSC^shSTC2^ (1×10^6^ per mouse) were injected via the tail vein after sensitization. Forty-eight hours post-challenge, ear and cervical lymph nodes (LN) samples were collected for analysis. **a** Representative photos of ears from mice in different groups. **b** Ear thickness (*n* = 6) was measured in the different experimental groups at the indicated times post-challenge. **c** Representative images of H&E-stained ear samples from mice in different groups, scale bar, 200 μm. **d** Representative plots of IFN-γ-producing CD8^+^ T cells within cervical LN cells, and bar chart showed the quantification. **e** The correlation analysis between the CD8^+^ effector T cells and the ear thickness of CHS mice. Data are presented as the mean ± SEM (*n* = 4). Statistically significant differences are indicated as follows: *p < 0.05 and **p < 0.01

To evaluate the roles of MSC-produced STC2 in T-cell-mediated immune responses in vivo, we induced CHS in mice using DNFB, injected the mice with MSCs (MSC^shNTC^ or MSC^shSTC2^) or saline via the tail vein, and measured ear swelling at 0, 24, 48, and 72 h after elicitation. Ear thickness increased progressively to a peak at 48 h post-challenge, and then decreased slightly by 72 h in saline-injected group. MSC^shNTC^-injected mice showed less swelling in DNFB-treated ears at 48 and 72 h compared with saline-injected mice. A similar trend was observed in MSC^shSTC2^-injected mice, but the ear swelling was more severe in MSC^shSTC2^-injected mice than in MSC^shNTC^-injected mice (Fig. 3a, b). Histopathological examination revealed that ear swelling and cellular infiltration were remarkable in the DNFB-treated ears of saline-injected mice; in contrast, these effects were not prominent in mice injected with MSC^shNTC^ or MSC^shSTC2^, with MSC^shNTC^-injected mice showing stronger improvements than MSC^shSTC2^-injected mice (Fig. 3c). These results demonstrate that knockdown of STC2 impairs the capacity of MSCs to alleviate CHS responses in mice.

### MSC^shSTC2^ mainly restored the CD8^+^ Tc1 cells in cervical lymph nodes

The CHS reaction occurs after cutaneous exposure to haptens. Antigen-presenting cells uptake haptens and express haptenated peptides, then they migrated to the draining lymph node, where they activate the pro-inflammatory cytokine producing effector T cells to mediate delayed-type hypersensitivity^29–31^. We postulated that MSC-produced STC2 might alleviate the effector T cells in inflamed cervical lymph nodes, which represent the regional draining lymph node in the murine CHS model. As expected, we found that the percentages of pro-inflammatory cytokine producing effector T cells, including IFN-γ-, TNF-α-, and IL-2-producing T cells, were remarkably increased in the cervical lymph nodes of CHS mice compared to controls (Fig. 3d, Fig. S7, Fig. S8), confirming that effector T cells were involved in this response. Importantly, MSC^shNTC^ treatment robustly decreased the percentages of both CD4^+^ (Fig. S7) and CD8^+^ effector T cells (Fig. 3d, Fig. S8). Consistent with our in vitro results, knockdown of STC2 in MSCs mainly decreased their ability to reduce the pro-inflammatory cytokine-producing CD8^+^ effector T cells in vivo (Fig. 3d, Fig. S8). The decrease in the IFN-γ-producing CD8^+^ T-cell subset was dramatically less in MSC^shSTC2^-injected mice than in MSC^shNTC^-injected mice (Fig. 3d). We further analyzed the correlation between the frequency of CD8^+^ effector T cells and the ear thickness of the CHS mice. As shown in Fig. 3e, the frequency of IFN-γ-producing CD8^+^ T cells was significantly positively correlated with the ear thickness of the CHS mice (*r* = 0.793, *p* < 0.01). However, the correlation was weak in TNF-α-producing CD8^+^ T cells (*r* = 0.618, *p* < 0.05), and none in IL-2-producing CD8^+^ T cells (*p* > 0.05). These results suggest that MSCs might alleviate CHS responses by reducing the pro-inflammatory cytokine producing effector T cells in regional draining lymph nodes, and that STC2 is involved in this process, mainly in reducing the CD8^+^ Tc1 cells.

### MSC^shSTC2^ mainly restored the infiltration of CD8^+^ Tc1 cells in inflammatory ear tissue

**Fig. 4:**
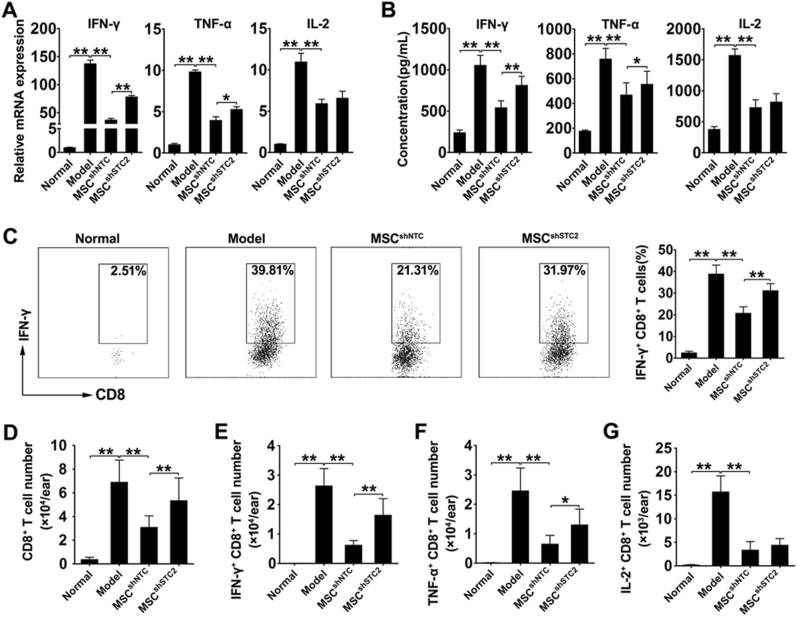
MSC-derived STC2 reduced the infiltration of CD8^+^ Tc 1 cells in inflamed ears. The level of IFN-γ, TNF-α, and IL-2 was assessed in inflammatory ears from mice in different groups. **a** Bar chart showed the mRNA level of IFN-γ, TNF-α, and IL-2 in inflammatory ears. **b** Bar chart showed the concentration of IFN-γ, TNF-α, and IL-2 in the ear homogenates. **c** Representative plots of alteration in percentages of IFN-γ-, TNF-α-, and IL-2-producing CD8^+^ T cells, and bar chart showed the quantification. **d**−**g** Bar chart showed the number of infiltrating total CD8^+^ T cells and IFN-γ-, TNF-α-, and IL-2-producing CD8^+^ T cells in inflammatory ears. Data are presented as the mean ± SEM (*n* = 5); *p < 0.05 and **p < 0.01

To further investigate the involvement STC2-mediated reduction of effector T cells in CHS response, we detected the levels of cytokine within the inflamed ear. RT-PCR results revealed that the expressions of IFN-γ, TNF-α, and IL-2 were significantly increased in the inflamed ears from CHS mice. MSC^shNTC^ treatment dramatically reduced their expressions, while MSC^shSTC2^ treatment showed a lower efficiency in decreasing IFN-γ, TNF-α, but not IL-2 (Fig. 4a). These results were further confirmed by the quantification of these cytokines in the ear homogenates by ELISA (Fig. 4b). Moreover, we evaluated the content of infiltrating T cells in the inflamed ears by flow cytometry. The results revealed the percentages of IFN-γ- (Fig. 4c, Fig. S9A), TNF-α- (Fig. S9B, Fig. S10A), and IL-2-producing T cells (Fig. S9C, Fig. S10B) were increased in both CD4^+^ and CD8^+^ T-cell subsets in inflamed ears from CHS mice, and MSC^shNTC^ treatment robustly reduced all these effector T cells. Interestingly, MSC^shSTC2^ treatment shows similar effects on IL-2-producing CD8^+^T cells and all CD4^+^ effector T cells compared with MSC^shNTC^ treatment, but a lower efficiency in decreasing IFN-γ-/TNF-α-producing CD8^+^T cells, especially the CD8^+^ Tc1 cells (Fig. 4c, Fig. S9A-C, Fig. S10A,B). Consistent with these alterations, the number of infiltrating total CD4^+^ and CD8^+^ T cells was increased in CHS mice and decreased in MSC^shNTC^ group. Knockdown of STC2 in MSCs mainly decreased their ability to reduce infiltrating CD8^+^ T cells, rather than CD4^+^ T cells (Fig. 4d, Fig. S9D). The alteration of the infiltrating CD8^+^ T cells was also demonstrated by the immunofluorescence staining of inflamed ear sections (Fig. S11). In addition, consistent with the alterations in cell percentages, we found that MSC-derived STC2 prone to reduce the number of infiltrating IFN-γ-/TNF-α-producing CD8^+^T cells, especially the CD8^+^Tc1 cells. As shown in Fig. 4e, IFN-γ-producing CD8^+^ T cells were rare in normal ear, but abundant in inflamed ears from CHS mice. Importantly, MSC^shNTC^ treatment dramatically decreased the number of infiltrating IFN-γ-producing CD8^+^ T cells in inflamed ears compared with that of saline-injected mice, whereas the number of infiltrating IFN-γ-producing CD8^+^ T cells in ears from MSC^STC2^-injected mice was not obviously altered. Taken together, these results further supported the idea that STC2 contributed to the capacity of MSCs to attenuate CHS chiefly via reducing the infiltration of CD8^+^ Tc1 cells.

### HO-1 activity is impaired in STC2-knockdown MSCs

**Fig. 5:**
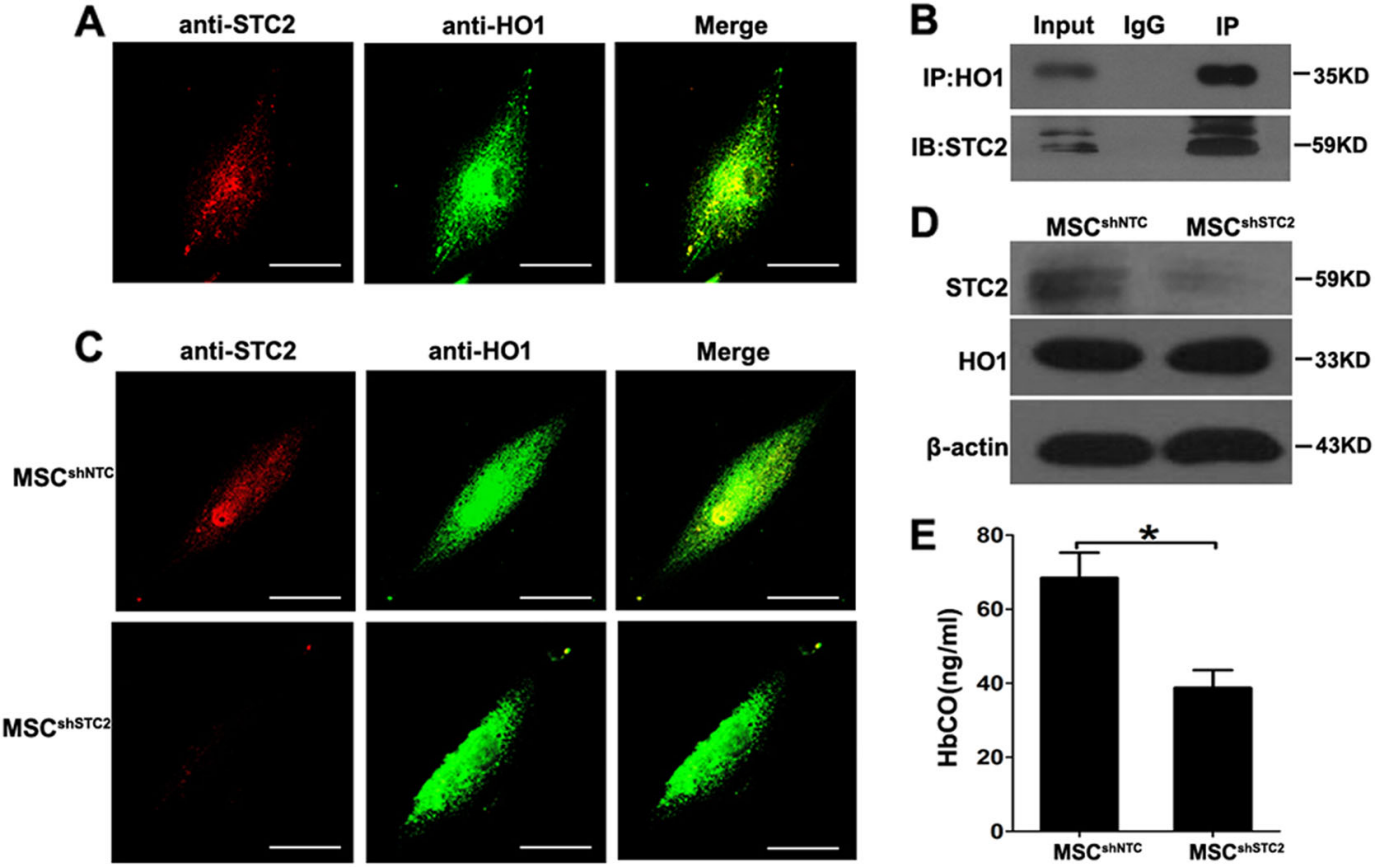
Silence of STC2 in MSCs impaired the activity of HO-1. **a** Cells were co-stained with anti-STC2 (red) and anti-HO-1 (green) and examined under immunofluorescence super-resolution microscopy. Scale bar, 200 μm. **b** Co-immunoprecipitation of HO-1 and STC2. Proteins were collected from MSCs and immunoprecipitated with anti-HO-1, and the immunoprecipitate was probed with anti-STC2. The controls included 5% of the input HO-1 and STC2 proteins (positive control) and IgG (negative control). The IP lane contains 100% total IP lysate. **c** MSC^shNTC^ or MSC^shSTC2^ were co-stained with anti-STC2 (red) and anti-HO-1 (green) and examined under immunofluorescence super-resolution microscopy. Scale bar, 200 μm. **d** Western blot analysis of STC2 and HO-1 proteins in MSC^shNTC^ and MSC^shSTC2^. **e** Culture supernatants of MSC^shNTC^ and MSC^shSTC2^ were treated with hemoglobin (50 μM/l), and CO levels were determined using an HbCO ELISA kit. Data are presented as the mean ± SEM (*n* = 4); *p < 0.05 and **p < 0.01

As STC2 is a secretory protein, we tested whether its free form could regulate CD8^+^ effector T cells, in a manner analogous to other soluble factors secreted by MSCs (e.g., TSG-6, TGF-β, and IL-10). Unexpectedly, the addition of rSTC2 failed to downregulate the pro-inflammatory cytokine producing CD8^+^ effector T cells (Fig. S12), neither the CD4^+^ effector T cells (Figs. S13), suggesting that MSC-derived STC2 may perform its immunoregulatory functions in an indirect manner. As STC2 was previously shown to form a complex with heme oxygenase-1 (HO-1) to degrade heme in COS-7 cells^32^, combined with HO-1 is an important mediator of the ability of MSCs to inhibit T-cell responses^33^, and downregulation of HO-1 in tumor increased the infiltration of cytotoxic CD8^+^ T cells^34^, we hypothesized that MSC-derived STC2 might reduce CD8^+^ Tc1 cells by affecting HO-1 activity. Indeed, immunofluorescence revealed that STC2 co-localized with HO-1 in MSCs (Fig. 5a), and co-immunoprecipitation confirmed that STC2 could interact with HO-1 in MSCs (Fig. 5b). Although knockdown of STC2 in MSCs did not alter the expression of HO-1, as assessed by immunofluorescence staining and western blotting (Fig. 5c, d), it significantly reduced the production of CO in a hemoglobin-degradation test (Fig. 5e). This suggests that HO-1 activity is impaired in STC2-knockdown MSCs. Thus, we speculated MSC-derived STC2 might reduce CD8^+^ Tc1 cells by regulating the activity of HO-1.

### The ability of MSC-derived STC2 to decrease CD8^+^ Tc1 cells involves HO-1

**Fig. 6:**
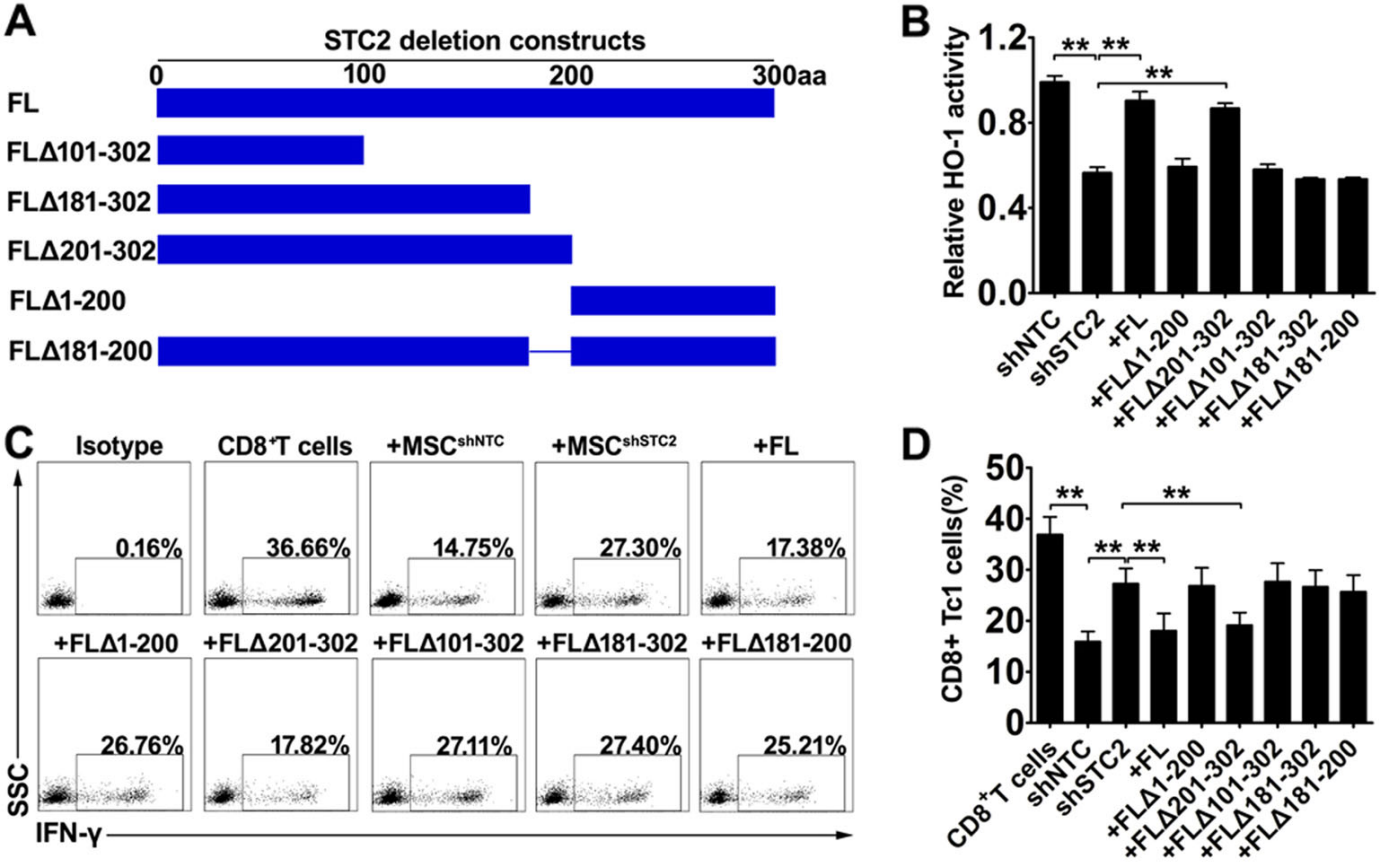
MSC-derived STC2 reduced the CD8^+^ Tc 1 cells by regulating the activity of HO-1. **a** The diagrammatic sketch of deletion constructs of human STC2. **b** The activities of HO-1 were evaluated by the production of CO using a hemoglobin-degradation test, and the bar chart showed the relative level of HO-1 activity. **c** Representative dot plots of the alterations of IFN-γ-producing CD8^+^ T cells were evaluated by flow cytometry, and bar chart showed the quantification (**d**). Data are presented as the mean ± SEM (*n* = 5); *p < 0.05 and **p < 0.01

A previous study reported that the binding site for HO-1 was located at amino acids 181–200 of STC2 ^32^. To further clarify the interaction of STC2 and HO-1 and their role in MSCs-mediated immunoregulation, we designed several deletion constructs of human STC2 (Fig. 6a), including FL (full-length, without deletion), FLΔ1–200 (deletion of amino acids 1–200), FLΔ101–302 (deletion of amino acids 151–302), FLΔ181–302 (deletion of amino acids 181–302), FLΔ201–302 (deletion of amino acids 201–302), FLΔ181–200 (deletion of amino acids 181–200). Then we transfected MSC^shSTC2^ with these constructs, and investigated their regulatory roles on HO-1 activity. As expected, knockdown of STC2 in MSCs impaired the activity of HO-1, and transfection of FL construct in MSC^shSTC2^ significantly rescued the HO-1 activity, indicating that STC2 in MSCs regulated the HO-1 activity. Importantly, the rescue of HO-1 activity was observed in the MSC^shSTC2^ transfected with FLΔ201–302 construct but not FLΔ1–200 construct. Considering the results that transfection of FLΔ101–302 construct or FLΔ181–302 construct failed to rescue the HO-1 activity, we speculated the interacting site might be located at the amino acids 181–200 of STC2. Indeed, the STC2 lacking amino acids 181–200 (FLΔ181–200 construct) failed to rescue the HO-1 activity (Fig. 6b). Taken together, STC2 could modulate the HO-1 activity, and this regulatory function of STC2 was mainly dependent on their interaction with HO-1 at the domain of amino acids 181–200.

To further investigate their role in MSC-mediated effects on the CD8^+^ Tc1 cells by STC2, the above transfected MSCs were co-cultured with CD3^+^ T cell and the alterations of IFN-γ-producing CD8^+^ T cells were detected by flow cytometry. As expected, transfection of FL or FLΔ201–302 construct in MSC^shSTC2^, but not FLΔ1–200, FLΔ101–302 or FLΔ181–302 construct, significantly restored the suppression of IFN-γ-producing CD8+ T cells, suggesting the amino acids 181–200 of STC2 is the functional domain (Fig. 6c, d). Indeed, the STC2 lacking amino acids 181–200 (FLΔ181–200 construct) failed to restore the suppression of IFN-γ-producing CD8^+^ T cells (Fig. 6c, d). Taken together, MSC-derived STC2 regulated the activity of HO-1, which likely contributed to MSC-mediated suppression of CD8^+^ Tc1 cells.

## Discussion

Previous studies showed that MSCs could inhibit the activation, proliferation, and pro-inflammatory cytokine production of conventional T cells^11,28,35^. However, the mechanisms underlying this regulation of T cells had not previously been explored in depth. Here, we report that MSC-derived STC2 plays an important role in inhibiting the pro-inflammatory cytokine secretion of T cells, but not suppressing T-cell proliferation or inducing Treg cells. Importantly, silencing the STC2 expression in MSCs abated their therapeutic effect on CHS in mice, restoring the generation and infiltration of CD8^+^ Tc1 cells. These novel findings suggest that STC2 may be an important mediator for the ability of MSCs to reduce the pro-inflammatory cytokines producing T cells, mainly the IFN-γ-producing CD8^+^ Tc1 cells.

The STC family consists of two members, STC1 and STC2, which are expressed in various human tissues^16^. STC1 is a peptide hormone that regulates calcium and phosphate homeostasis, and has been shown to have various biological effects, such as protecting retinal ganglion cells by inhibiting apoptosis and oxidative damage, and promoting the survival of lung cancer cells by uncoupling oxidative phosphorylation^23^. STC1 also acts as an anti-inflammatory protein, as it reportedly inhibits the response of macrophages to chemoattractants^36^ and regulates inflammation in lung injury^24^. These findings led us to speculate that STC1 could potentially contribute to the ability of MSCs to regulate the immune response. However, we found that STC1 was expressed at only a very low level in MSCs. A previous study found that MSC-derived STC1 is involved in the ability of these cells to correct the inappropriate epithelial−mesenchyme relationship in pulmonary fibrosis^37^. This coupled with our findings and a report that MSCs could express STC1 upon TGF-β1 or H_2_O_2_ stimulation, suggest that STC1 may be inducible in MSCs.

STC2, which is a homolog of STC1, is a stress-responsive protein believed to act as a target of the oxidative stress response to protect cells from apoptosis^26^. STC2 has also been shown to reduce TNF-α production in LPS-stimulated BV2 cells^26^, and to enhance survival and long-term stemness in MSCs^38^. Here, we report for the first time that STC2 appears to contribute to the immunomodulatory properties of MSCs by mediating their ability to downregulate the pro-inflammatory cytokines producing T cells, mainly the IFN-γ-producing CD8^+^ Tc1 cells.

CHS, which is a T-cell-mediated cutaneous inflammatory reaction to haptens, requires the action of pro-inflammatory-cytokine-producing T cells, such as CD4^+^ Th1 and CD8^+^ Type 1 cytotoxic T cells^29,31^. Notably, the murine responded to dinitrochlorobenzene (DNCB) or dinitrofluorobenzene (DNFB), commonly used models of ACD, were dependent on CD8^+^ T cells, mainly the IFN-γ-producing CD8^+^ T cells^5–7^. The analogous finding was reported in patients with contact dermatitis that responded to urushiol (poison ivy)^39^. Here, we show that MSC-derived STC2 can downregulate the percentage of CD8^+^ effector T cells in vitro and in the CHS mouse model in vivo, and that it has particularly strong effects on CD8^+^ Tc1 cells. These actions may contribute significantly to the ability of MSCs to alleviate inflammatory reactions in CHS.

Although STC2 is known to regulate calcium transport, phosphate transport, cell metabolism, and cytoprotection, little is known about its ability to modulate immune responses. STC2 is a secreted glycoprotein that may have autocrine or paracrine functions. However, we found that the proportions of the pro-inflammatory cytokines producing T cells were not affected by the addition of rSTC2 to our MSC/T cell co-culture system. This led us to speculate that STC2 might modulate intracellular signaling events that are responsible for the immunomodulatory functions of MSCs. Previous intracellular interaction studies showed that STC2 could interact with HO-1 to form a eukaryotic “stressosome” in COS-7 cells^32^. Similar with this report, we found that STC2 co-localized with HO-1 in MSCs. HO-1, which catalyzes the degradation of heme and releases CO as a main degradation product^40^, is key to the MSC-mediated suppression of alloactivated T cells^41^. Interestingly, we found that STC2 knockdown did not alter the expression of HO-1, but rather decreased the production of CO in a heme-treated system. Furthermore, transfection of STC2 full length, but not STC2 lacking amino acids 181–200, rescues the HO-1 activity in MSC^shSTC2^. These suggest that STC2 can modulate the HO-1 activity, and this regulatory function of STC2 was mainly dependent on their interaction with HO-1 at the domain of amino acids 181–200.

Studies have shown that HO-1 activity is important for the various HO-1-mediated immunomodulatory functions of MSCs. For example, the HO-1 activity inhibitor, Tin (Sn) protoporphyrin-IX (SnPP), abolished the ability of MSCs to suppress T cells^42^. In addition, CO has been shown to alter IFN-γ signaling and significantly decrease the production of TNF, thereby generating anti-inflammatory effects^43,44^. Recent studies have demonstrated that HO-1 and CO play regulatory roles in acute inflammatory states and MSC-mediated inhibition of pro-inflammatory cytokines that produced by T cells^45,46^. Furthermore, the infiltration of cytotoxic CD8^+^ T cells in tumor was regulated by HO-1^34^. Similarly, we found that knockdown of STC2 in MSCs impaired the activity of HO-1, which weaken their capacity in decreasing the CD8^+^ Tc 1 cells.

In summary, our results collectively show that MSC-derived STC2 can reduce the CD8^+^ Tc1 cells, and that this effect contributes to the ability of injected MSCs to alleviate the inflammatory reaction in CHS mice. Mechanistically, STC2 regulates the activity of HO-1, which is a main controller of the immunomodulatory effects of MSCs on T cells. This is the first time that STC2 has been shown to affect an immune reaction that regulates effector T cells. Moreover, our findings suggest that MSC-derived STC2 might represent a promising novel therapeutic target for the treatment of CD8^+^ T-cell-mediated immune disorders.

## Materials and methods

### MSCs isolation, expansion, and characterization

MSCs isolated from bone marrow samples obtained from healthy donors followed Declaration of Helsinki protocols with informed consent, and the protocol was approved by the relevant Ethics Review Board prior to initiation. Briefly, mononuclear cells were obtained by Ficoll-Hypaque (GE, http://www3.gehealthcare.com) density gradient centrifugation and seeded to 75-cm^2^ flasks (CellBIND, Corning). After 3 days of culture, the medium was replaced and nonadherent cells were discarded. At 70–80% confluence, the cells were harvested by trypsin and cultured at 1×10^4^ cells/cm^2^ in 75-cm^2^ flasks. At passage 5, the surface expressions of CD29, CD34, CD44, CD45, CD73, CD90, CD105, and CD166 were detected by flow cytometry (BD Biosciences). Also at passage 5, the multipotency of the isolated MSCs was confirmed by their in vitro differentiation to adipocytes and osteocytes as described in our previous report^15^.

### Animals

BALB/c mice (male, 6–8 weeks old) were purchased from the Guangdong Medical Laboratory Animal Center, China (Certification number: 0055283). The animals were maintained in an approved animal facility under specific pathogen-free conditions. They were housed three or four per stainless-steel wire cage, without bedding, under controlled temperature (18–26 °C) and humidity (50 ± 20%), and with a 12 h light/12 h dark cycle. All animals were provided a standard diet and acidified water ad libitum. All animal care and experiments were performed under protocols approved by the Institutional Animal Care and Use Committee of the Zhongshan Medical School at Sun Yat-Sen University.

### Contact hypersensitivity model

CHS reactions were induced in mice with DNFB. Briefly, 25 μl of 0.5% DNFB (Sigma) in acetone/olive oil (4:1) was applied evenly to a shaved hind flank for 2 consecutive days. On day 5, sensitized mice were challenged on the right ear by application of 10 μl of 0.25% DNFB solution in acetone/olive oil (4:1). An identical amount of acetone/olive oil (4:1) was administered to the left ear. MSCs were intravenously injected via the tail vein on the day of challenge. The control group received phosphate-buffered saline. Ear thickness was measured at 24, 48, and 72 h post-challenge by individuals blinded to the treatment status. In some studies, mice were sacrificed 48 h post-challenge, and ear samples and cervical lymph nodes were harvested, then cells were isolated, stained, and the content of total T cells and the proinflammatory cytokine-producing T cells by flow cytometry was evaluated.

### RNA interference

For STC2 knockdown, MSCs were transduced with a lentiviral vector encoding the shRNA or an insert-free vector (negative control, designated “shNTC”), the shRNA sequence is presented in Table S1. Lentiviruses were produced in transfected 293T cells. MSCs at passage 5 or 8 were seeded to six-well plates and transduced using the X-tremeGENE HP reagent (Roche).

### Co-culture experiments

Human peripheral blood mononuclear cells were collected from healthy donors, and CD3^+^ T cells, CD3^+^CD8^+^ T cells, and CD3^+^CD4^+^ T cells were purified by the BD Influx (BD Bioscience). MSCs were seeded to 24-well flat-bottom plates. After 24 h, CD3^+^ T cells, CD3^+^CD8^+^ T cells, or CD3^+^CD4^+^ T cells were separately added to the MSCs, and co-culture experiments were performed for 3 days.

### Flow cytometry

Cell-surface markers and intracellular cytokines were analyzed using a Gallios Flow Cytometer (Beckman Coulter, Fullerton, CA, USA) following standard protocols. The data were analyzed using the Kaluza software packages (Beckman Coulter). The utilized antibodies included anti-CD8-Pacific blue, anti-CD3-FITC, anti-CD3-V450, anti-IFN-γ-PE-Cy7, anti-IL2-FITC, and anti-TNF-α-APC-Cy7 from BD Bioscience, and anti-CD4-FITC, anti-CD25-PE, and anti-FoxP3-APC from eBioscience (San Diego, CA, http://www.ebioscience.com).

### Cytokine assays

T cells were cultured with or without MSCs (5:1 ratio) for 3 days. During the last 6 h of incubation, PMA (50 ng/ml) and ionomycin (500 ng/ml) were added to the culture system, and brefeldin A (BFA; 10 μg/ml) was used to inhibit cytokine secretion (all from Sigma Aldrich). IFN-γ, TNF-α, and IL-2 were analyzed by flow cytometry.

### Proliferation assays

Purified T cells were stained with 5 μM 5-(and-6)-carboxyfluorescein diacetate succinimidyl ester (CFSE, CellTrace; Invitrogen, Carlsbad, CA, USA). The CFSE-labeled T cells (2×10^5^ cells/well) were incubated with or without MSCs in 24-well plates treated with PHA (5 μg/ml). After 4 days, the percentage of proliferating T cells was detected by CFSE dilution.

### Treg assays

The percentage of CD4^+^CD25^+^Foxp3^+^ Tregs was evaluated using a Human Regulatory T-Cell Staining Kit 2 (eBioscience) according to the manufacturer’s instructions.

### Immunofluorescence

MSCs were grown on 24-well plates and cultured with conditioned medium as indicated. After 48 h, the cells were fixed in 3.7% formaldehyde for 20 min, permeabilized by 0.2% Triton X-100 for 10 min. After treatment with 10% goat serum for 30 min, primary antibodies and species-specific secondary antibodies conjugated with either Alexa Fluor 594 or Alexa Fluor 488, and nuclei were counter-stained with DAPI (4′,6-diamidino-2-phenylindole) for 10 min. Images were acquired using an LSM780 confocal microscope (Zeiss).

For direct immunofluorescence staining in ear sections, 5 μm cryo-sections were fixed in 4% paraformaldehyde (PFA), blocked in 5% FBS plus 5% rat serum and incubated with the following antibodies: Alexa Fluor® 594 anti-mouse CD8a anti-body (Biolegend), nuclei were counterstained using DAPI. Images were acquired using an LSM780 confocal microscope (Zeiss). CD8^+^T cells in the ear epidermis were quantified in 3–5 images per specimen using ImageJ software.

### RNA isolation and quantitative real-time PCR

Total RNA from cells and tissues were extracted with the TRIzol reagent (Invitrogen, Carlsbad, CA), and reverse transcription (RT) was performed using a RevertAid First Strand cDNA Synthesis Kit (Thermo Scientific, Vilnius, Lithuania). The cDNA thus obtained was subjected to real-time PCR with the SYBR Green reagent (Roche, Indianapolis, IN) using the primers listed in Table S2.

### Western blot assays

MSCs were grown in six-well plates and lysed with lysis buffer (62.5 mM Tris-HCl, pH 6.8, 2% SDS, 10% glycerol, 0.02% bromophenol blue, and 50 mM DTT). Proteins were separated by 10% sulfate-polyacrylamide gel electrophoresis, transferred to a polyvinylidene fluoride membrane, blocked with TBS/T containing 5% nonfat dry milk, and probed with specific primary antibodies against STC1, STC2, and HO1 (all from Abcam).

### Oligonucleotides and plasmid construction

In transient transfection experiments, human STC2 cDNA was cloned into the PKD-Flag-IRES2-Puro expression vector through *Eco*R1 and *Mlu*I1 digestion sites to produce C-terminally FLAG-tagged STC2. Mutated STC2 cDNA fragments were amplified with the above construct as template, and cloned into the same expression vector at the same digestion sites. The oligonucleotides used as PCR primers are listed in Table S3.

### ELISA assay

CO binds about 200 times more strongly and quickly to hemoglobin (Hb) than oxygen, yielding the HbCO complex, which can be detected as a means to measure the level of CO in the culture medium^47,48^. Briefly, cells were plated to six-well plates and incubated at 37 °C for 35 h. Human hemoglobin (50 μM/l) was added, the plates were incubated for an additional 1 h, and equal volumes of cell culture supernatant were collected. HbCO was quantified using a human HbCO ELISA kit (Elabscience Technology, Wuhan, China) according to the manufacturer’s protocol. The results were obtained at 450 nm using a microplate reader (Tecan Trading AG, Switzerland). For tissue cytokines detection, ears were homogenized in PBS (0.01 M, pH = 7.4) containing protease, and phosphatase inhibitors, the ratio tissue/PBS is 1:9. After incubation at 4 °C and clarification of the samples by centrifugation, IFN-γ, TNF-α, and IL-2 were quantified by ELISA kits from Elabscience according to the manufacturer’s instructions. Cytokine concentrations were normalized to protein concentration of the lysates.

### Statistical analysis

All results are expressed as the mean ± SEM. Statistical comparisons were made using a two-tailed Student’s *t* test (between two groups) or a one-way ANOVA (for multi-group comparisons). Changes in ear thickness were compared using a repeated measure ANOVA. *p* < 0.05 was considered to represent a significant difference. Analysis and graphing were performed using the Prism 5.01 software package (GraphPad, San Diego, CA).

## Electronic supplementary material

Supplementary Figures and Tables

Supplementary figure legends
