# Supplementary material for: Stanniocalcin-2 contributes to mesenchymal stromal cells attenuating murine contact hypersensitivity mainly via reducing CD8+ Tc1 cells
Source: Cell Death Dis. 2018 May 10;9(5):548. doi: 10.1038/s41419-018-0614-x (PMC5945630; doi:10.1038/s41419-018-0614-x)
Supplement: Supplementary file 1 — Supplementary Figures and Tables [file 41419_2018_614_MOESM1_ESM.docx]

**1. Supplementary Figures**

**
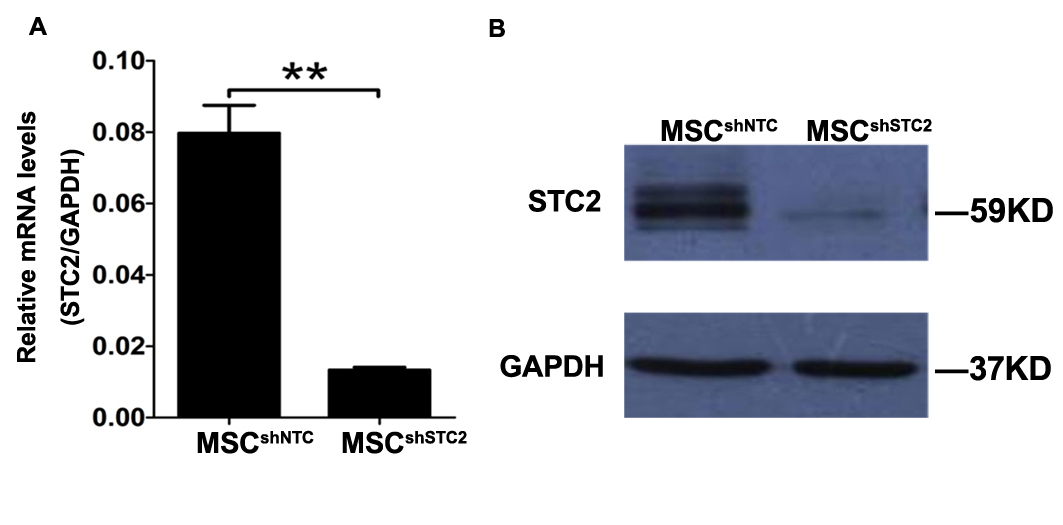
**

**Figure S1. Knockdown of STC2 in MSCs by retroviral transfection.**

(A). The efficiency of shRNA-mediated down-regulation of STC2 was assessed by the RNA level, and normalized with respect to the expression of GAPDH. The bar graphs indicate the mean ± SEM, and statistically significant differences are indicated as follows: * *p* < 0.05 and ** *p* < 0.01. (B) Western blot analysis of the STC2 knockdown in whole-cell lysates of MSCs. The expression of GAPDH was used as a control.


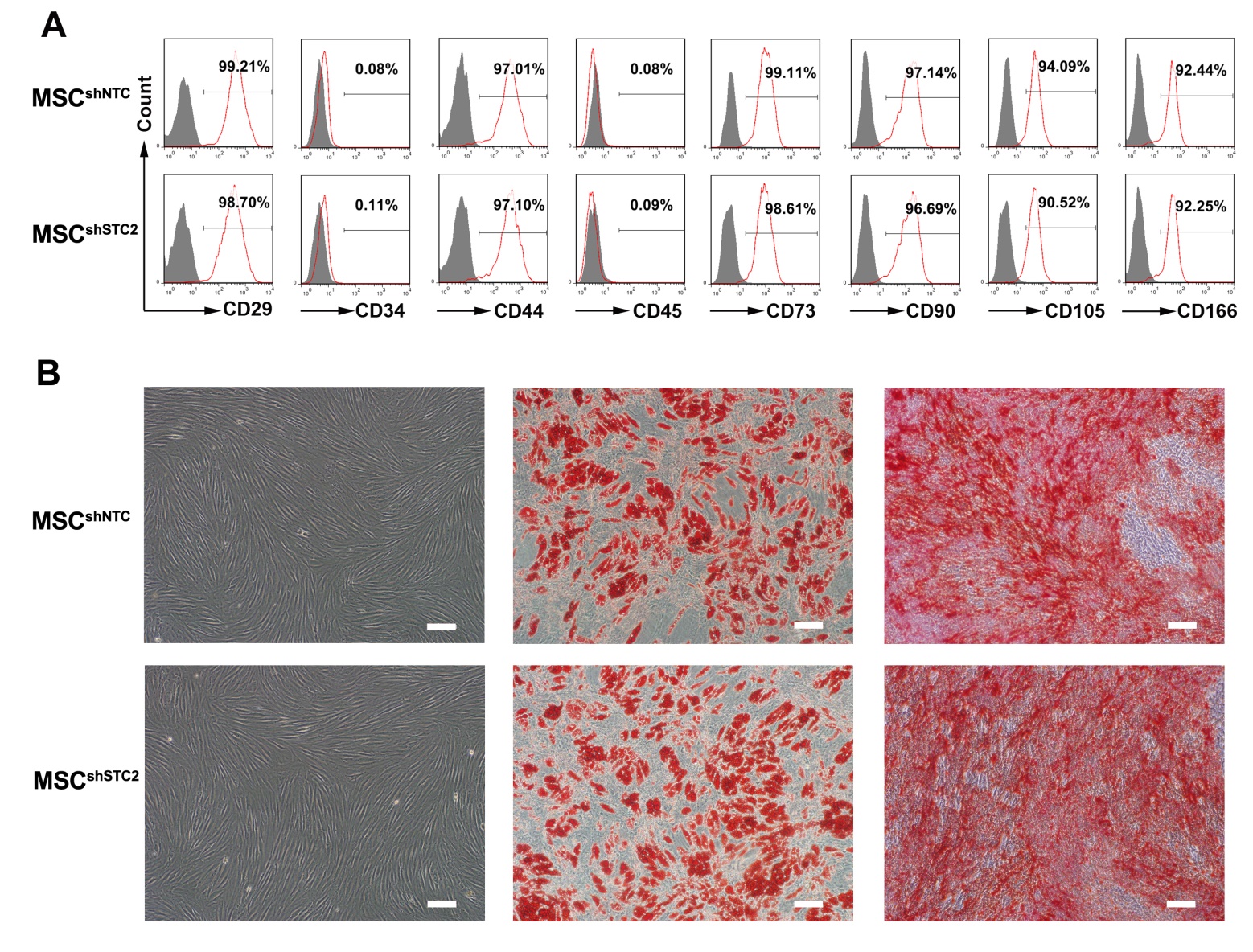


**Figure S2. The characteristics of MSCs are not altered by STC2 knockdown.**

The characteristics of MSCs were compared between MSC^shNTC^ and MSC^shSTC2^. (A) Representative plots of cell-surface markers, CD29, CD34, CD44, CD73, CD90, CD105, and CD166 on MSC^shNTC^ and MSC^shSTC2^. (B) Cell shape, adipogenic differentiation, and osteogenic differentiation of MSC^shNTC^ and MSC^shSTC2^. Scale bar, 200 μm.

**.**


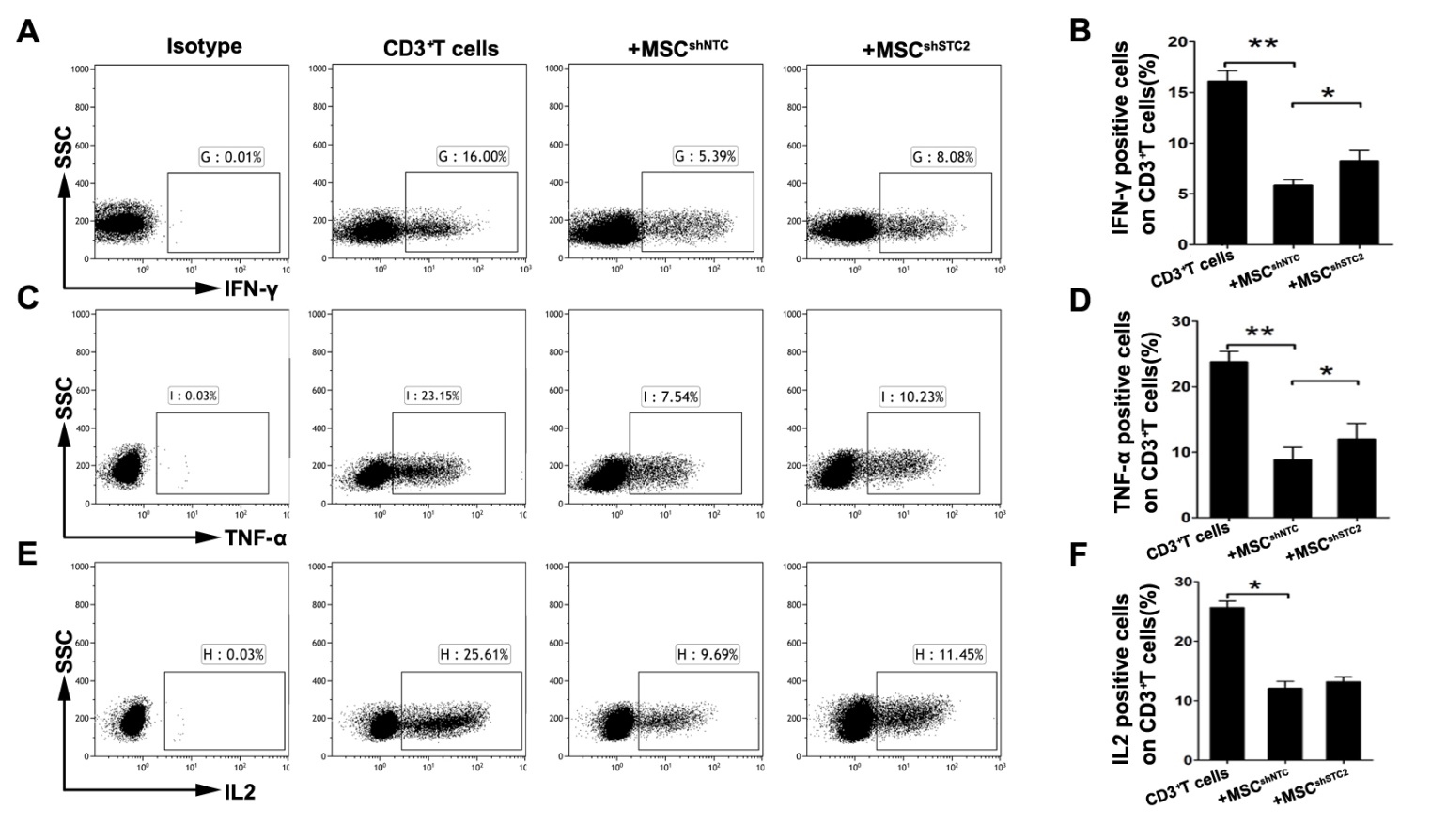


**Figure S3. MSCs down-regulate the pro-inflammatory cytokine producing T cells partially through STC2 in vitro.**

CD3^+^ T cells were co-cultured with MSC^shNTC^ or MSC^shSTC2^ for 3 days, and flow cytometry was used to assess IFN-γ-producing T cells (A, B), TNF-α-producing T cells (C, D), and IL-2-producing T cells (E, F). The bar graphs indicate the mean ± SEM. Statistically significant differences are indicated as follows: * *p* < 0.05 and ** *p* < 0.01.


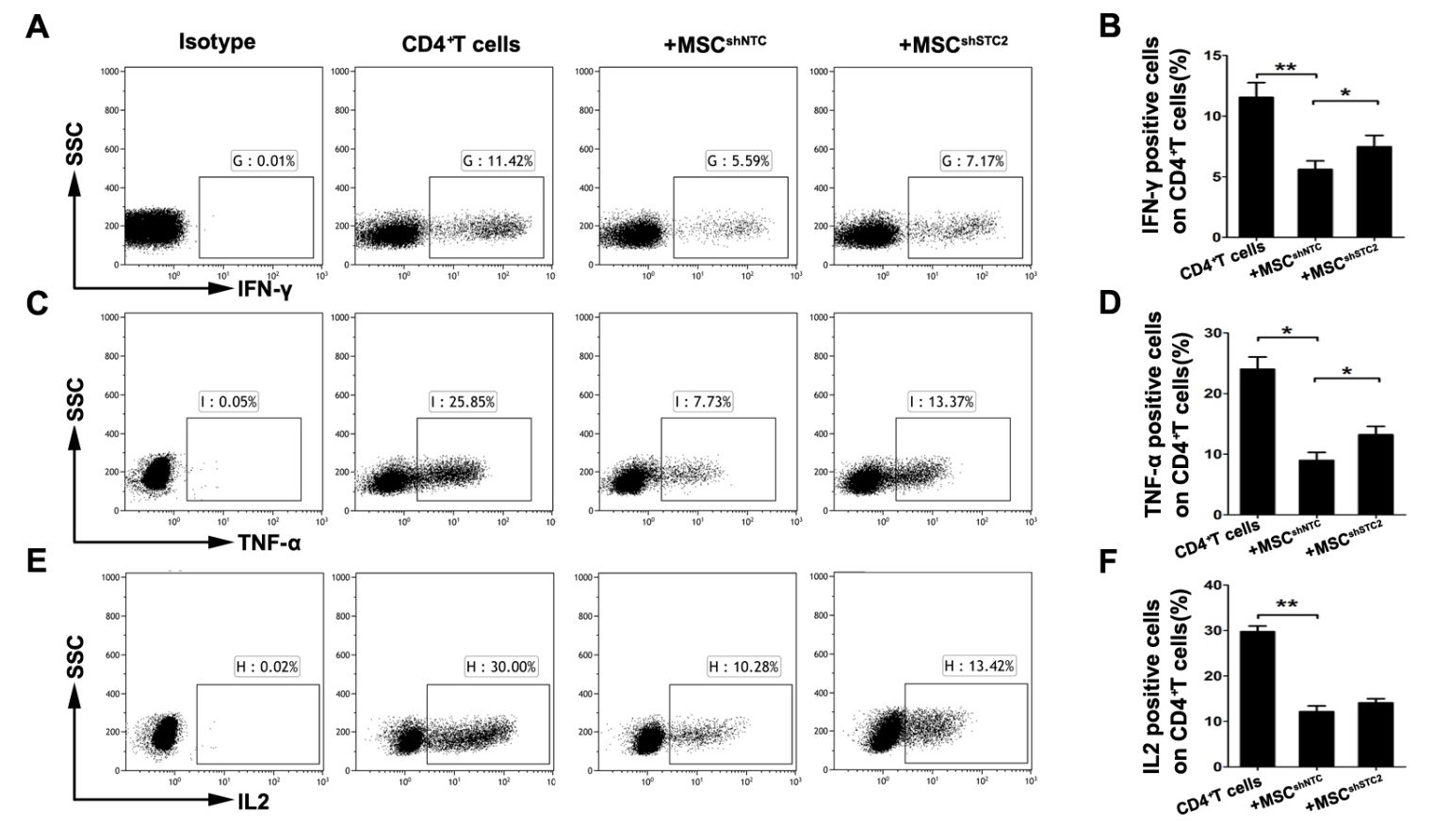


**Figure S4. MSCs down-regulate the pro-inflammatory cytokine producing CD4+ T cells partially through STC2 in vitro.**

CD3^+^CD4^+^ T cells were co-cultured with MSC^shNTC^ or MSC^shSTC2^ for 3 days, and flow cytometry was used to assess IFN-γ-producing T cells (A, B), TNF-α-producing T cells (C, D), and IL-2-producing T cells (E, F). The bar graphs indicate the mean ± SEM. Statistically significant differences are indicated as follows: * *p* < 0.05 and ** *p* < 0.01.


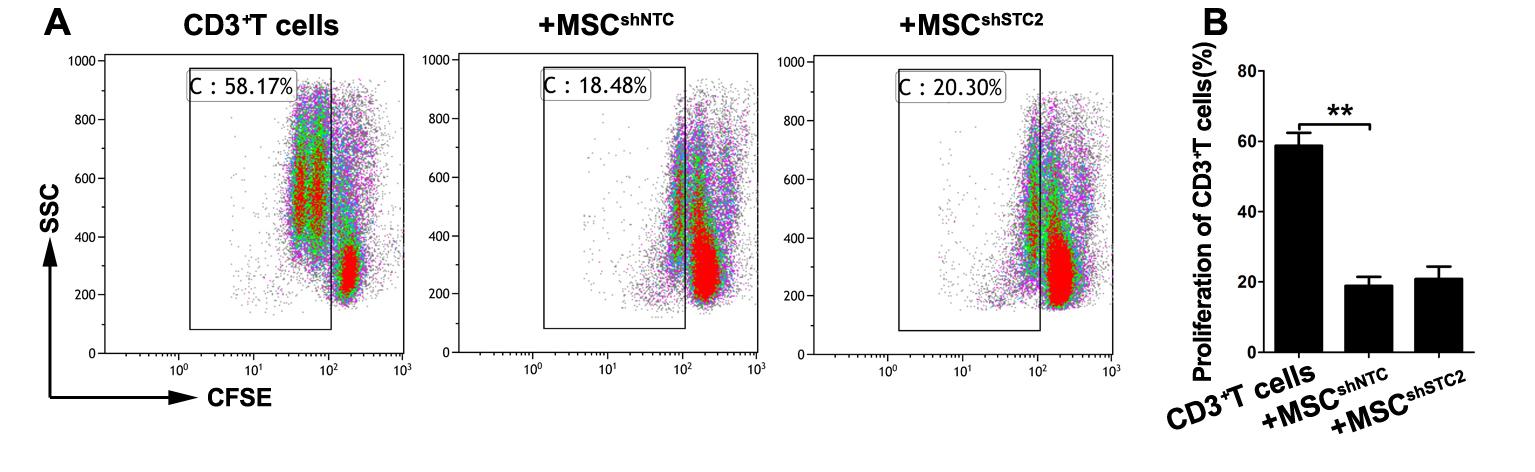


**Figure S5. STC2 has little effect on MSC-mediated T cell proliferation inhibition.**

The T-cell proliferative response was evaluated by the CFSE dilution. (A) Representative flow cytometry dot plots of T cell proliferation; (B) The bar graphs indicate the mean ± SEM. Statistically significant differences are indicated as follows: * *p*< 0.05 and ** *p*< 0.01.


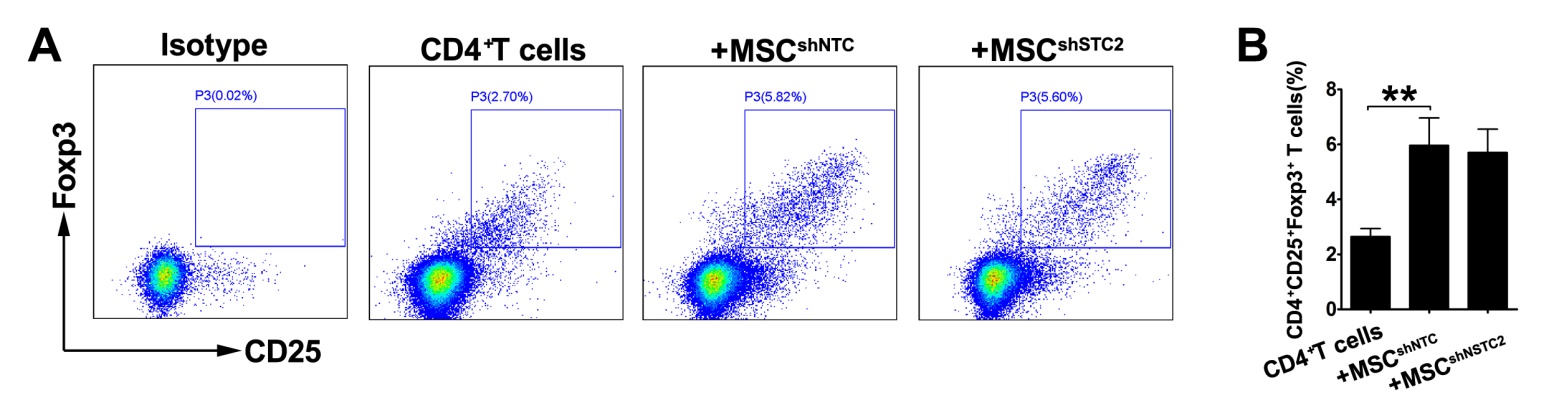


**Figure S6. STC2 has little effect on MSC-mediated Treg cell induction.**

(A) T cells were co-cultured with MSCs for 3 days, then the expression of CD4^+^CD25^+^FoxP3^+^Treg cells was assessed by flow cytometry. (B) The bar graphs indicate the mean ± SEM. Statistically significant differences are indicated as follows: * *p*< 0.05 and ** *p*< 0.01.


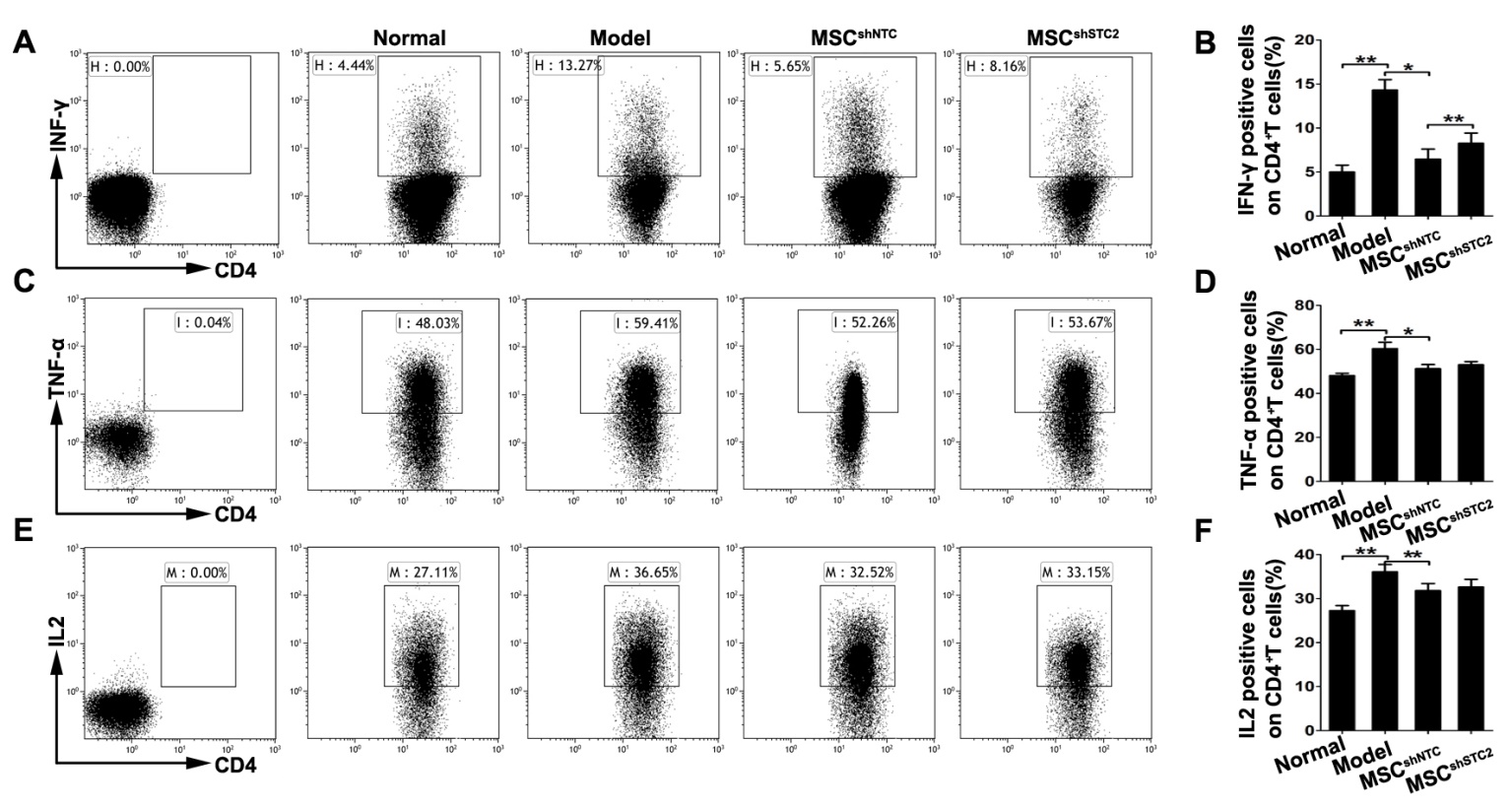


**Figure S7. MSCs reduce the CD4^+^ effector T cells in cervical lymph nodes.**

The pro-inflammatory cytokine producing CD4^+^ T effector T cells in cervical LN were detected. Representative plots of IFN-γ-, TNF-α-, IL-2-producing CD4^+^ T cells within cervical LN cells (A, C, E), and bar chart showed the quantification (B, D, F). Data are presented as the mean ± SEM (n=4); **p*< 0.05 and ***p*< 0.01.


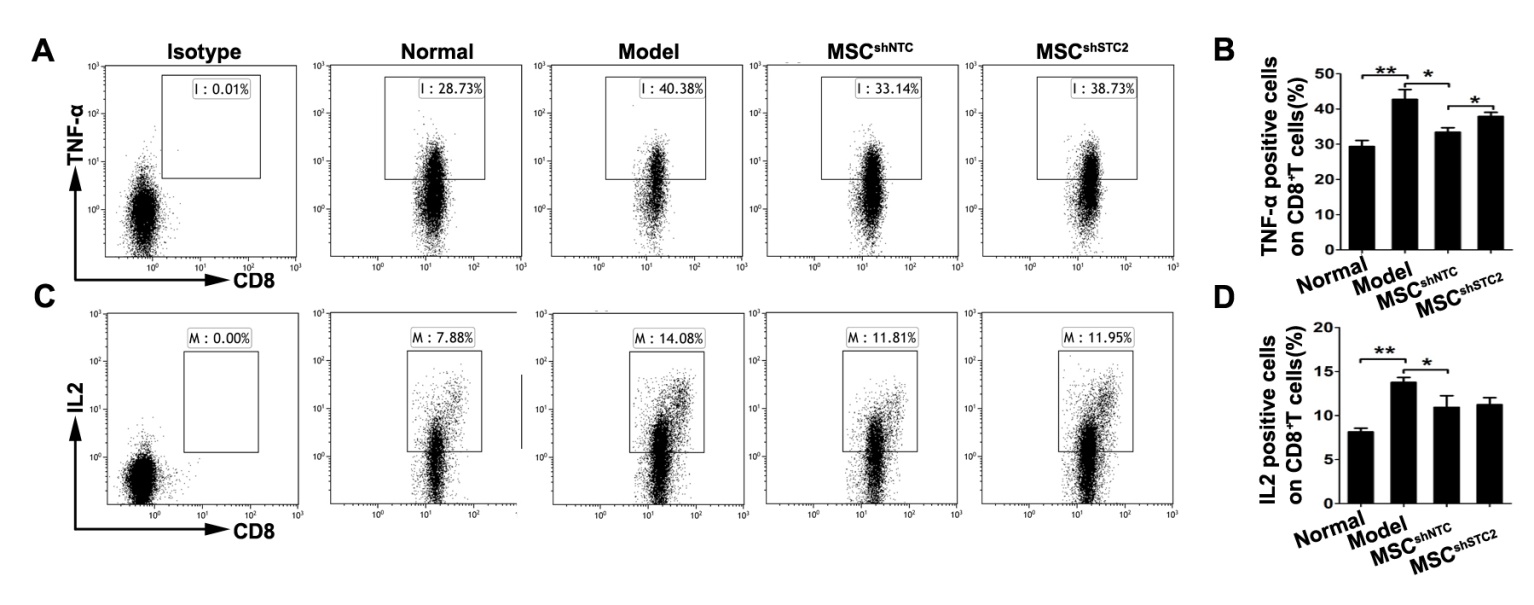


**Figure S8. MSCs reduce the TNF-α- and IL-2-producing CD8^+^ effector T cells in cervical lymph nodes.**

The pro-inflammatory cytokine producing CD8^+^ T effector T cells in cervical LN were detected. Representative plots of TNF-α- and IL-2-producing CD4^+^ T cells within cervical LN cells (A, C), and bar chart showed the quantification (B, D). Data are presented as the mean ± SEM (n=4); **p*< 0.05 and ***p*< 0.01.


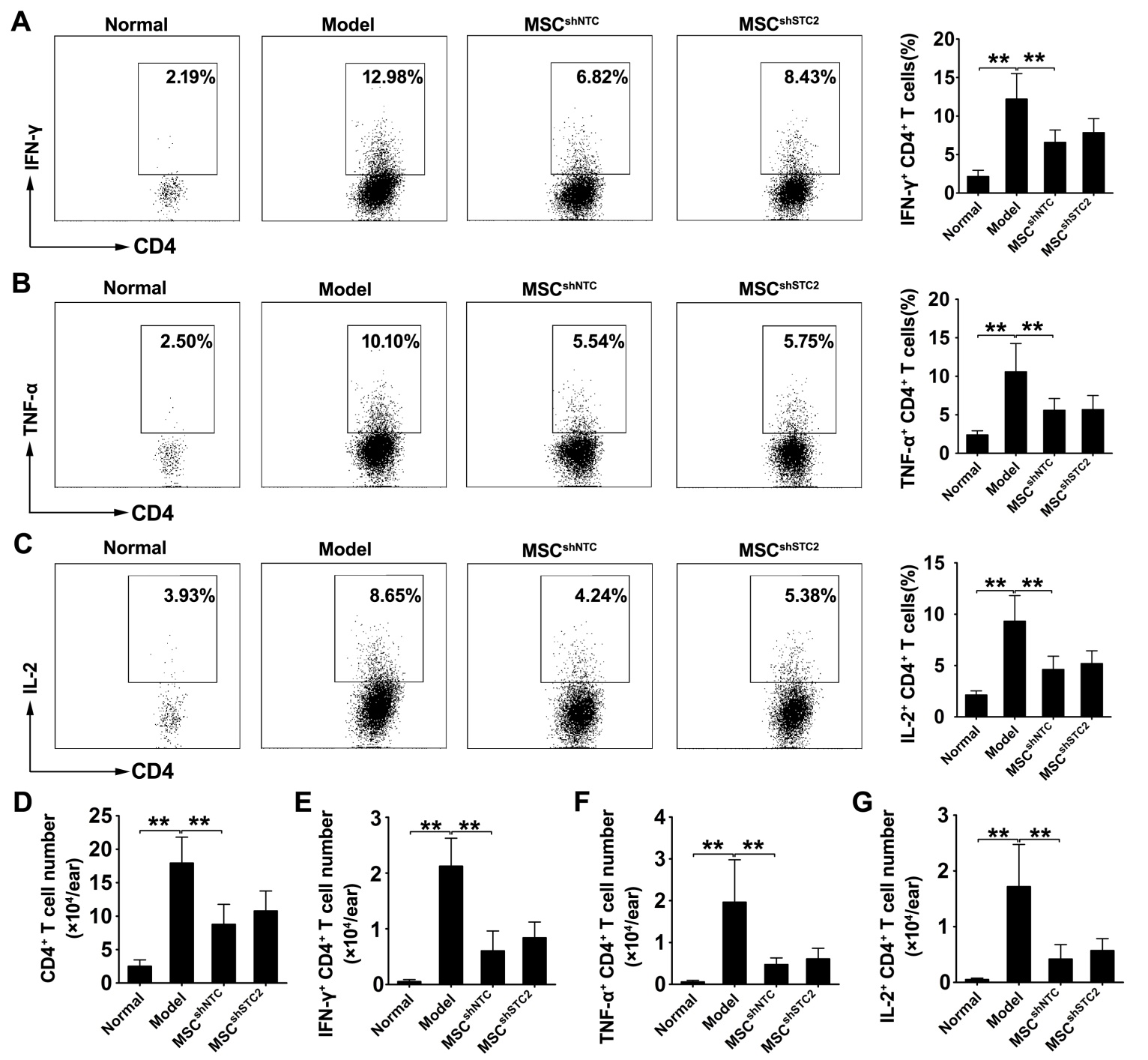


**Figure S9. MSCs reduce the CD4^+^ effector T cells in inflamed ears.**

The pro-inflammatory cytokine producing CD4^+^ T effector T cells in inflamed ears were detected. Representative plots of IFN-γ-, TNF-α-, IL-2-producing CD4^+^ T cells within inflamed ear isolated cells (A-C), and bar chart showed the quantification both in frequency (A-C) and cell number (D-G). Data are presented as the mean ± SEM (n=5); **p*< 0.05 and ***p*< 0.01.


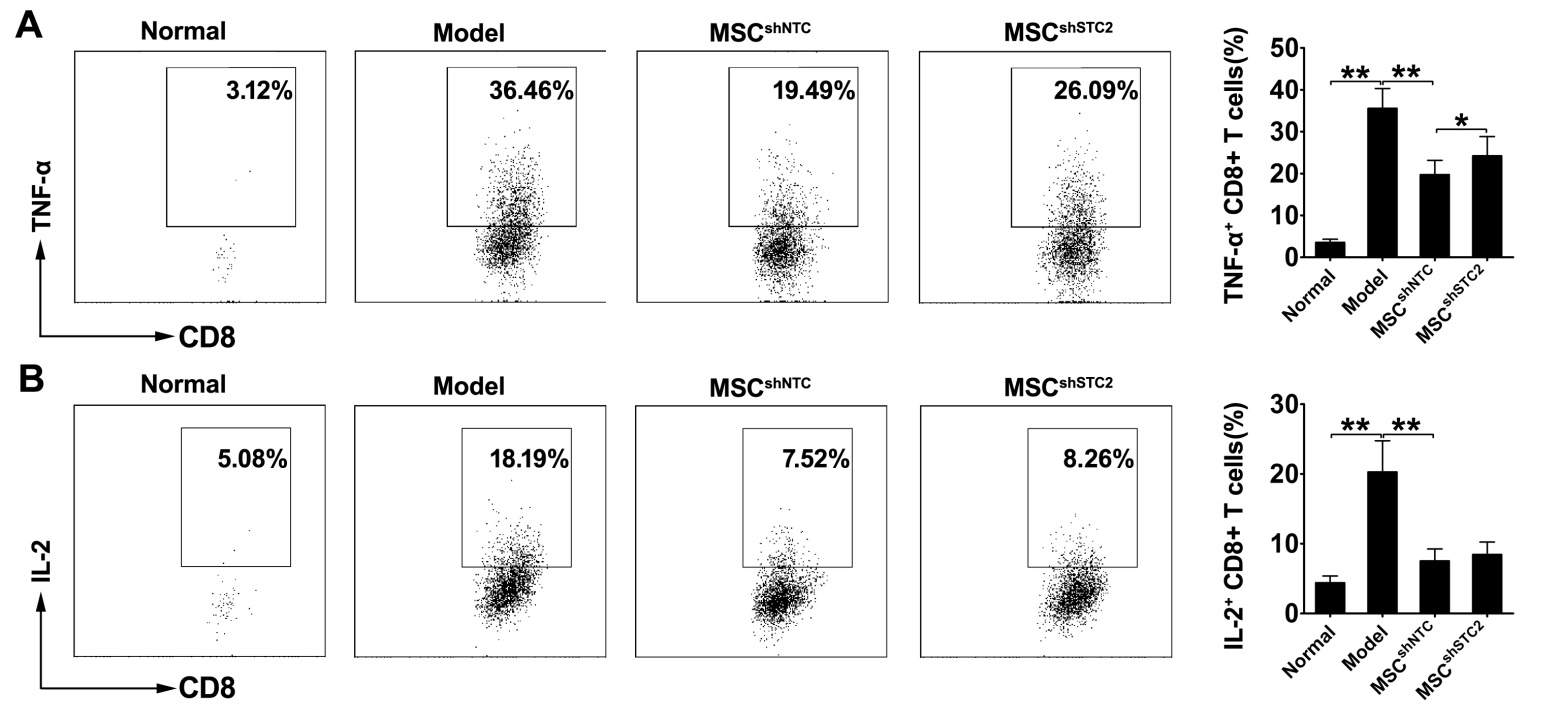


**Figure S10. MSCs reduce the TNF-α- and IL-2-producing CD8^+^ effector T cells in inflamed ears.**

The pro-inflammatory cytokine producing CD8^+^ T effector T cells in inflamed ears were detected. Representative plots of TNF-α- and IL-2-producing CD4^+^ T cells within inflamed ears isolated cells (A, B), and bar chart showed the quantification . Data are presented as the mean ± SEM (n=5); **p*< 0.05 and ***p*< 0.01.


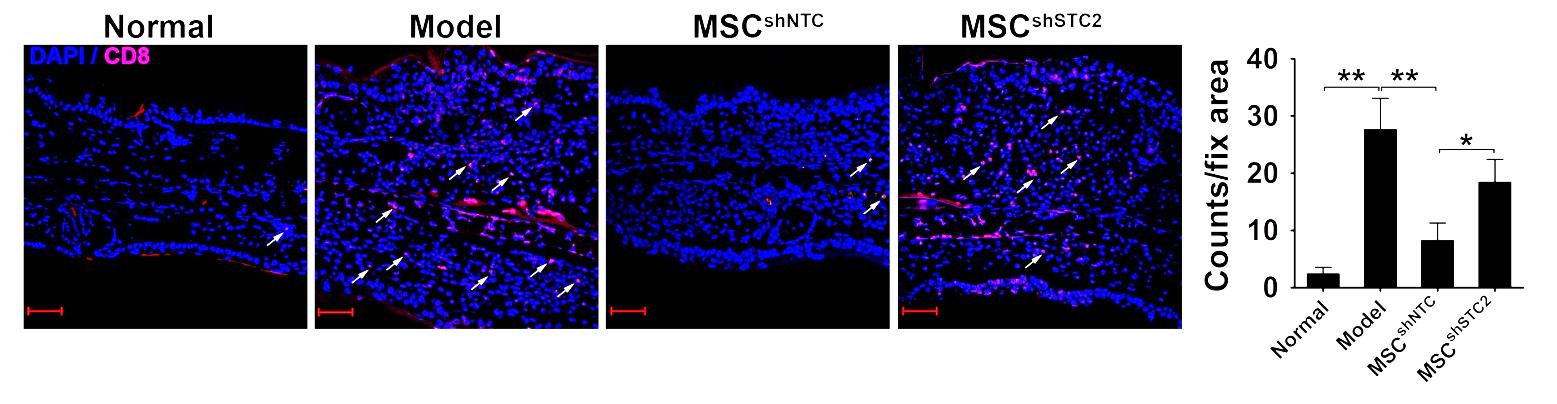


**Figure S11. MSCs reduce the CD8^+^ T cells in inflamed ears.**

Representative immunofluorescence images of CD8^+^ T cells that infiltrating in the inflamed ear, and bar chart showed the quantification. Scale bar, 50 μm. **p*< 0.05 and ***p*< 0.01.


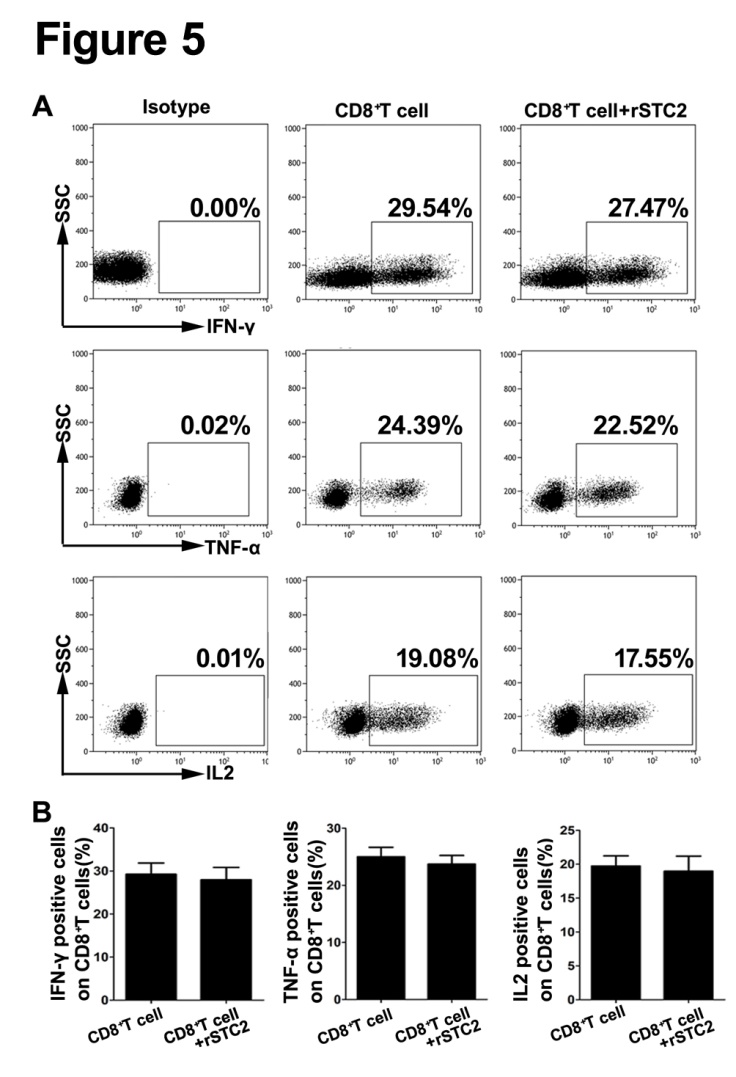


**Figure S12. The rSTC2 protein does not alter the pro-inflammatory cytokine producing CD8^+^ effector T cells.**

CD3^+^CD8^+^ T cells were treated without or with recombinant human STC2 (rSTC2, 50 ng/ml), and the productions of IFN-γ, TNF-α, and IL-2 were assessed. (A) Representative plots of alteration in percentages of IFN-γ-, TNF-α- and IL-2-producing CD8^+^ T cells, and bar chart showed the quantification (B). Data are presented as the mean ± SEM (n=4); * *p* < 0.05 and ** *p*< 0.01.


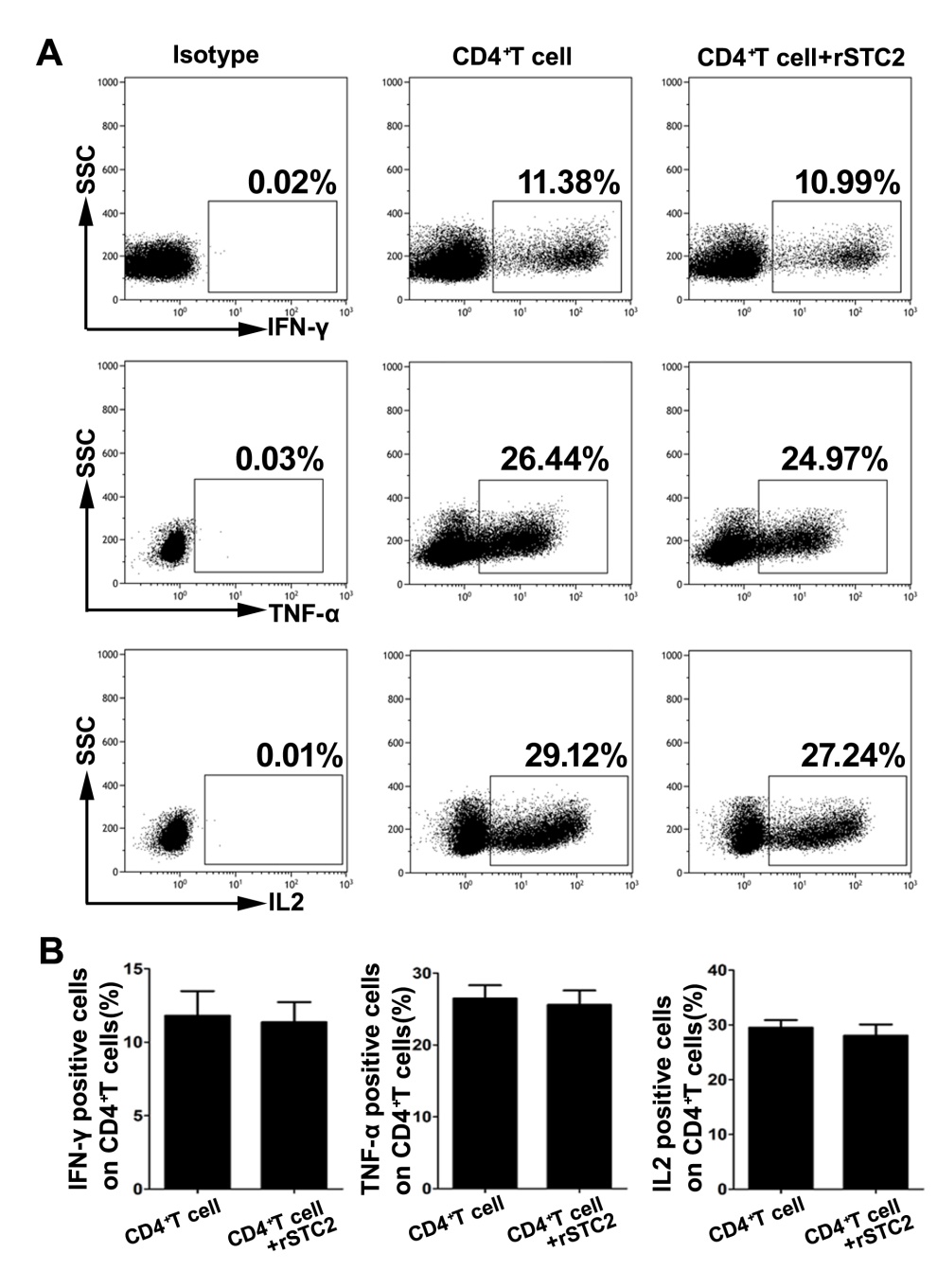


**Figure S13. The rSTC2 protein does not alter the pro-inflammatory cytokine producing CD4^+^ effector T cells.**

CD3^+^CD4^+^ T cells were treated without or with recombinant human STC2 (rSTC2, 50 ng/ml), and the productions of IFN-γ, TNF-α, and IL-2 were assessed. (A) Representative plots of alteration in percentages of IFN-γ-, TNF-α- and IL-2-producing CD4^+^ T cells, and bar chart showed the quantification (B). Data are presented as the mean ± SEM (n=4); * *p* < 0.05 and ** *p*< 0.01.

**2. Supplementary Tables**

**Table S1. STC2 shRNA sequence used to generate lentivious plasmid for RNA silencing**

|  | Oligonucleotide | | |
| --- | --- | --- | --- |
| Forward | | 5’-CCGGAAAAGACGAACAGTCTGAGTACTCGAGTACTCAGACTGTTCGTCTTTTTTTTTG-3’ | |
| Reverse | | | 5’-AATTCAAAAAAAAAGACGAACAGTCTGAGTACTCGAGTACTCAGACTGTTCGTCTTTT-3’ |

**Table S2. Primer list for** **RT-PCR assay.**

| Gene | Sequence | |
| --- | --- | --- |
| human *GAPDH* | | F: GCAAATTCCATGGCACCG |
|  | | R: TCGCCCCACTTGATTTTGG |
| human *STC1* | | F: TGGTTCGTTGCCTCAACAGT |
|  | | R: GAAGACCTTGGAGGTGACCC |
| human *STC2* | | F: CTGTCCCTGCAGAATACAGC |
|  | | R: CAAATTTTCCAGCGTTGTG |
| mice *TNF* | | F: GGTGCCTATGTCTCAGCCTCTT |
|  | | R: GCCATAGAACTGATGAGAGGGAG |
| mice *IFNG* | | F: GACAATCAGGCCATCAGCAAC |
|  | | R: AGCTCATTGAATGCTTGGCG |
| mice *IL2* | | F: ATGAACTTGGACCTCTGCGG |
|  | | R: GTCCACCACAGTTGCTGACT |
| mice *18S rRNA* | | F: GTAACCCGTTGAACCCCATT |
|  | | R: CCATCCAATCGGTAGTAGCG |

**Table S3. Primer list for mutated STC2 cDNA fragments construction.**

| construction | Sequence |
| --- | --- |
| pKD-hSTC2 | F: tccaggcctaagcttacgcgtATGTGTGCCGAGCGGCTG |
|  | R: gtcgtccttgtagtcgaattcCCTCCGGATATCAGAATACTCAGAC |
| pKD-hSTC2(Δ201-302) | F: tccaggcctaagcttacgcgtATGTGTGCCGAGCGGCTG |
|  | R: gtcgtccttgtagtcgaattcGTTCTGCTCACACTGAACCTGCA |
| pKD-hSTC2(Δ181-302) | F: tccaggcctaagcttacgcgtATGTGTGCCGAGCGGCTG |
|  | R: gtcgtccttgtagtcgaattcGGTCAGCAGCAAGTTCACGAG |
| pKD-hSTC2(Δ101-302) | F: tccaggcctaagcttacgcgtATGTGTGCCGAGCGGCTG |
|  | R: gtcgtccttgtagtcgaattcCTTGCCCTGGGCATCAAAT |
| pKD-hSTC2(Δ1-200) | F: tccaggcctaagcttacgcgtTGGGGAAGCCTGTGCTCC |
|  | R: gtcgtccttgtagtcgaattcCCTCCGGATATCAGAATACTCAGAC |
| pKD-hSTC2 (Δ181-200) | F: aacttgctgctgaccgaattcTGGGGAAGCCTGTGCTCC |
|  | R: gtcgtccttgtagtcgaattcCCTCCGGATATCAGAATACTCAGAC |
